# Genome-wide association study reveals a patatin-like lipase relating to the reduction of seed oil content in *Brassica napus*

**DOI:** 10.1186/s12870-020-02774-w

**Published:** 2021-01-06

**Authors:** Haoyi Wang, Qian Wang, Haksong Pak, Tao Yan, Mingxun Chen, Xiaoyang Chen, Dezhi Wu, Lixi Jiang

**Affiliations:** 1 Institute of Crop Science, Zhejiang University, Yu-Hang-Tang Road 866, Hangzhou, 310058 China; 2 State Key Laboratory of Crop Stress Biology for Arid Areas and College of Agronomy, Northwest A&F University, Yangling, 712100 Shaanxi China; 3 Institute of Crop Science, Jinhua Academy of Agricultural Sciences, 828 Shuanglong Nan, Jinhua, 321017 China

**Keywords:** Seed oil content, Lipase, Patatin, GWAS, Rapeseed, Lipid degradation, RNA-seq analysis

## Abstract

**Background:**

Rapeseed (*Brassica napus* L.) is an important oil crop world-widely cultivated, and seed oil content (SOC) is one of the most important traits for rapeseed. To increase SOC, many efforts for promoting the function of genes on lipid biosynthesis pathway have been previously made. However, seed oil formation is a dynamic balance between lipid synthesis and breakdown. It is, therefore, also reasonable to weaken or eliminate the function of genes involved in lipid degradation for a higher final SOC.

**Results:**

We applied a genome-wide association study (GWAS) on SOC in a collection of 290 core germplasm accessions. A total of 2,705,480 high-quality SNPs were used in the GWAS, and we identified *BnaC07g30920D*, a *patatin-like lipase* (*PTL*) gene, that was associated with SOC. In particular, six single-nucleotide-polymorphisms (SNPs) in the promoter region of *BnaC07g30920D* were associated with the significant reduction of SOC, leading to a 4.7–6.2% reduction of SOCs. We performed in silico analysis to show a total of 40 *PTLs*, which were divided into four clades, evenly distributed on the A and C subgenomes of *Brassica napus*. RNA-seq analysis unveiled that *BnPTLs* were preferentially expressed in reproductive tissues especially maturing seeds.

**Conclusions:**

We identified *BnaC07g30920D*, a *BnPTL* gene, that was associated with SOC using GWAS and performed in silico analysis of 40 *PTLs* in *Brassica napus*. The results enrich our knowledge about the SOC formation in rapeseed and facilitate the future study in functional characterization of *BnPTL* genes.

**Supplementary Information:**

The online version contains supplementary material available at 10.1186/s12870-020-02774-w.

## Background

Rapeseed (*Brassica napus* L., AACC, 2n = 38) is the third-largest source of vegetable oil in the world after soybean and palm [1]. Seed oil content (SOC) is one of the most important traits in rapeseed. Overall, the SOC among rapeseed varieties varies from 26 to 50% [2]. It is an important goal for rapeseed breeders to have varieties with high SOC.

Seed oil accumulation is a dynamic balance between lipid biosynthesis and breakdown. Lipid biosynthesis in plant seeds is activated after seed embryo formation and it involves a series of pathways, such as carbohydrate metabolism, fatty acids (FAs) elongation, triacylglycerol (TAG) synthesis and oil-body formation [3]. In brief, the synthesis of lipids in seeds can be roughly divided into three stages. First, those 16C-18C FAs including palmitic acid (C16:0), stearic acid (C18:0), and oleic acid (C18:1) are synthesized in plastids using pyruvate from glycolysis as the primary substrate. Second, the 16C-18C FAs are transported to the endoplasmic reticulum, where they are catalyzed by a series of enzymes for the elongation and desaturation of the carbon chains. Finally, TAGs are synthesized based on FAs and glycerol, and stored in 1–2 μm oil bodies [3–8]. The transcription factors (TFs) such as *LEAFY COTYLEDON1* (*LEC1*), *LEC2*, *ABA INSENSITIVE3*, *FUSCA3* and *WRINKLED1* are key regulators of seed FA biosynthesis and are expressed in seeds. Their expressions overlap various developmental stages [6, 9].

The accumulation of lipids in rapeseed is not a unilateral synthesis process but a dynamic balance between anabolism and catabolism. Seed lipids reduce at the late stage of seed maturation at an approximate rate of 10% on average [10]. Oil degradation is catalyzed by a variety of lipases that hydrolyze TAG and set FAs free [11]. The free FAs are unstable and subjected to further degradation for acetyl-CoA in a process known as beta-oxidation taking place in glyoxysomes. The acetyl-CoA is further degraded into 4-carbon compounds in the so-called glyoxylate cycle occurring in peroxisomes [1, 12–14].

It is estimated that the reduction of SOC at the late maturation stage of rapeseed results in a loss of about 20 million tons of oil per year [10]. A previous study revealed that five genes, namely *BnSFAR1* to *BnSFAR5* belonging to the GDSL family are involved in the decomposition of lipids in developing seeds of rapeseed, and thus reduced SOC [15]. However, GDSL lipase could not be the only type of lipase that play roles in seed lipid decomposition.

Patatin is a single lipolytic acyl-hydrolase with broad substrate specificity, firstly found in potato tubers in 1970 [16]. Instead of GXSXG or GDSL motif, the active site of the patatin domain consists of a distinct Ser-Asp catalytic dyad [17]. Studies indicated that plant patatin-related enzymes are involved in diverse biofunctions, including plant responses to auxin, elicitors or pathogens, and abiotic stresses and lipid mobilization during seed germination [18–21]. Many patatin-like lipases (PTLs) such as SUGAR-DEPENDENT1 (SDP1) act as phospholipases or TAG lipases [18, 22, 23]. The functions of most PTLs remain little understood.

In the past decades, quantitative trait locus (QTL) mapping unearthed plenty of information on the molecular mechanism controlling the formation of total FAs [24, 25] and FA species [26, 27] in rapeseed. More recently, genome-wide association studies (GWAS) combined with linkage disequilibrium (LD) mapping emerged as a more effective tool in identifying candidate genes that control agronomic and quality traits in a large genetic population [28, 29]. The rapid development of genome sequencing technologies enabled the identification of single nucleotide polymorphisms (SNPs) in a huge quantity [28–32]. LD mapping which indicates the degree of correlation between significant SNPs and candidate genes is a population-based survey technology for a precise QTL localization [28, 29, 33–35].

To better understand the genetic control of SOC in a polyploid genome of rapeseed, we performed GWAS on SOC in a collection of 290 core germplasm accessions representing a total of 991 genetic accessions originated from 39 countries of the world. We identified *BnaC07g30920D*, a *BnPTL* gene, that was significantly associated with SOC. We analyzed the expression of *Bna.C07.PTL* in various tissues, and performed in silico analysis to show an overall distribution of the *PTL* family in the rapeseed genome.

## Results

### Phenotypic variation of SOC

We measured the SOC of the 290 core accessions (Table S1) at two locations, namely, Changxing (CX) and Jinhua (JH), in 2018 and 2019, respectively. The overall statistics of the SOC were listed in Table 1. The SOC ranged from 29.80 to 54.52% in CX and from 35.09 to 56.55% in JH, with an average of 43.25 and 47.98%, respectively (Table 1). The coefficient of variations (CVs) for CX and JH were nearly identical, indicating a similar degree of SOC variation at these two locations (Table 1). The SOCs in both locations were approximately normal-distributed, and the SOCs of most accessions ranged between 40 and 50% (Figure S1). Analysis of variance (ANOVA) was performed. It was found that both genotype and environment had significant effects on SOC (*P* < 0.01) (Table 1).

**Table 1:** The overall statistics of phenotypic variation and analysis of variance (ANOVA) of SOC in the 290-accession core collection

Trait	Locations	Mean ± SD (%)	Range (%)	Skewness	Kurtosis	CV (%)	Genotype	Environment
SOC	CX	43.25 ± 2.86	29.80–54.52	−0.386	2.383	6.6	**	**
	JH	47.98 ± 3.21	35.09–56.55	−0.876	1.81	6.7		

Note: SD standard deviation, CV coefficient of variation, ** (*P* < 0.01)

### Identification of a *BnPTL* gene significantly associated with SOC

**Fig. 1:**
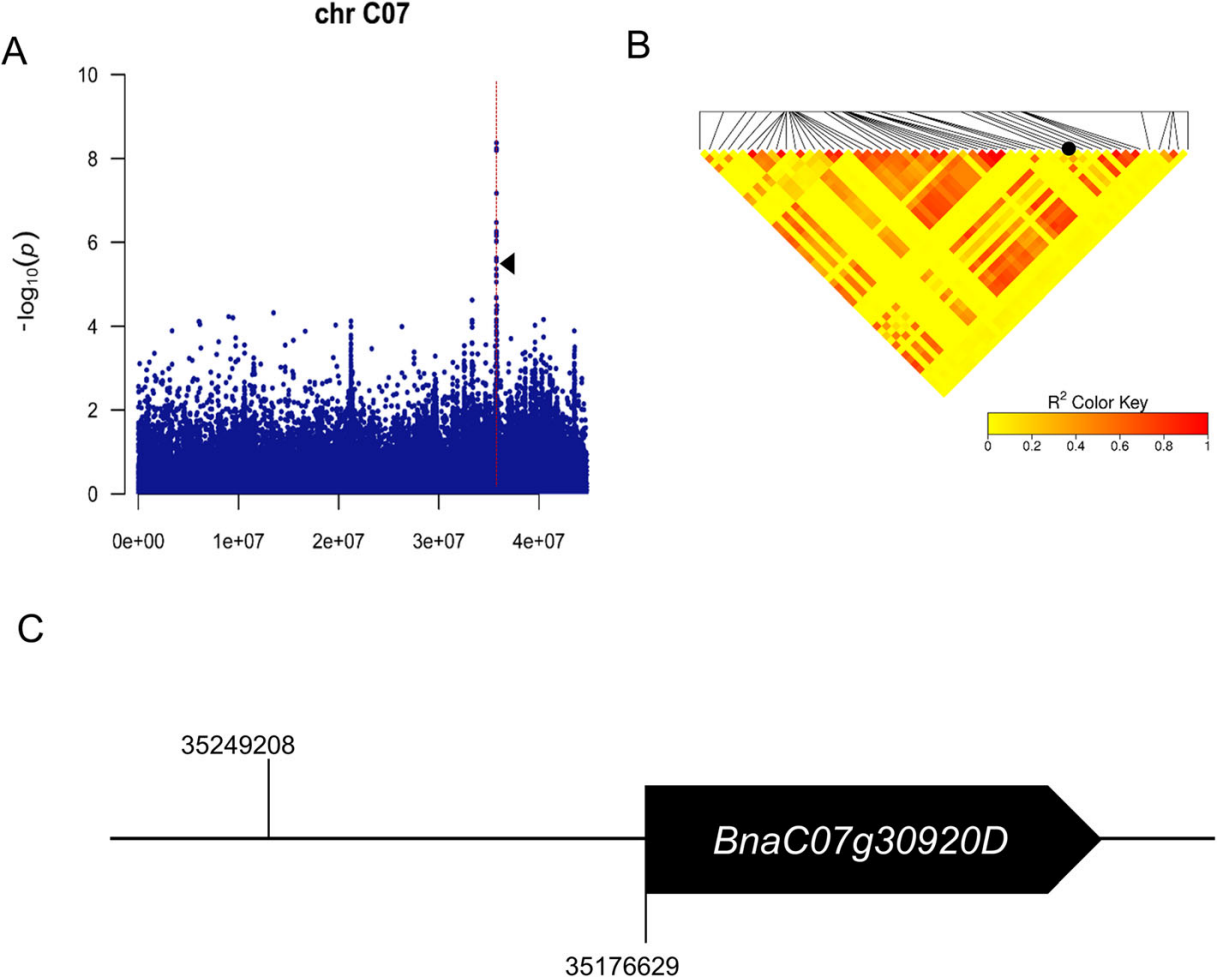
*BnaC07g30920D* is associated with SOC reduction in rapeseed. **a** Local Manhattan plot showing the SNPs at the peak region (indicated by the dotted red line) on Chr.C07 that are significantly (−log_10_*p* > 5) associated with SOC. The small black triangle indicates the SNP, ChrC07_35249208, associated with *BnaC07g30920D*. **b** Haploblock showing markers in linkage disequilibrium (LD). The black dot on the LD heat map indicates the position of the representative SNP, ChrC07_35249208. The linkage degrees between each of the two positions and between the SNPs and the candidate gene *BnaC07g30920D* are shown by the color keys indicating *r*^*2*^ values. **c** A sketch magnifying the relative distance between the significant SNP associated with SOC and *BnaC07g30920D*

Using 2,705,480 SNPs (MAF > 0.05, missing rate < 0.5), we performed GWAS on SOC in a population including 290 accessions at CX and JH. The Manhattan Plots show that most significantly associated SNPs (*p* < 10^− 5^) were mapped on chromosome C07 (Chr.C07) and random chromosome An (Chr.Ann) for the experiment at CX (Figure S2A), and were on Chr.A08 and Chr.C07 for the experiment at JH (Figure S2B), respectively. The quantile-quantile (Q-Q) plots showed that there was a significant correlation between SOCs and genotypes by natural selection (Figure S3). In light of the results from both environments, the SNPs located between 35.25 Mbp and 35.79 Mbp on Chr.C07 were taken for further investigation (Figure S2, Table S2). A total of 31 and 22 SNP signals on Chr.C07 were significantly associated with SOC at CX and JH, respectively (Table S2), suggesting 50 and 34 genes on Chr.C07 in CX and JH, respectively, that might be responsible for SOC variations (Table S2). Of these, *BnaC07g30920D*, annotated as a patatin-like phospholipase, was a putative ortholog of *At1g33270* encoding an adiponutrin (ATGL)-like protein. The SNP, ChrC07_35249208, on Chr.C07 was linked (R^2^ = 0.68) with *BnaC07g30920D* (Fig. 1), indicating their linkage disequilibrium. There were 55 SNPs at *BnaC07g30920D*, including 51 SNPs located on the 5′-end regulatory region, 2 SNPs on the coding sequence (CDS) region, and 2 SNPs within introns (Table S3).

**Fig. 2:**
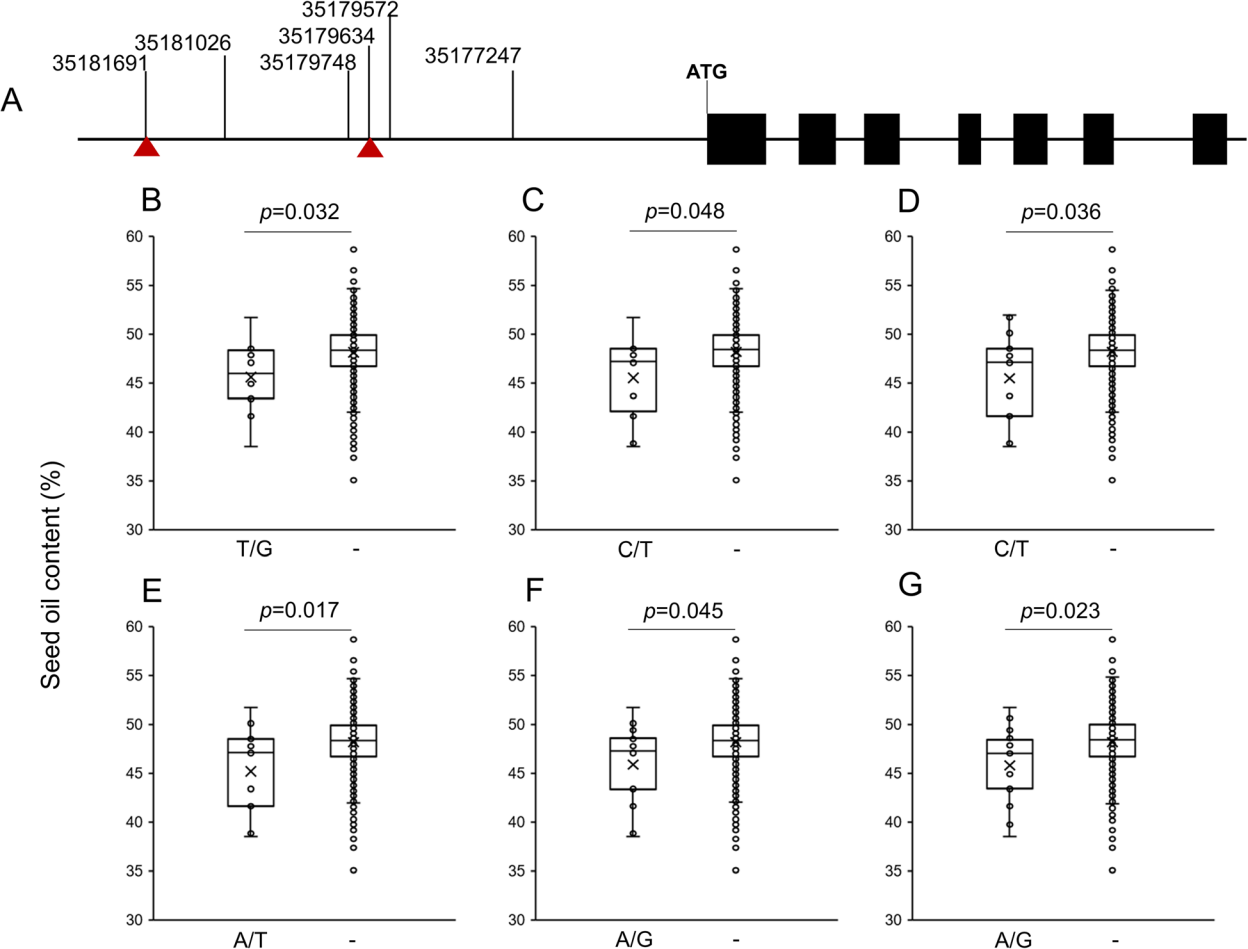
The allelic changes in *BnaC07g30920D* alter SOC among 290 accessions. **a** A sketch showing the structure of *BnaC07g30920D* and the position of the six alleles associated with SOC. Red triangles point to the positions of the cis-elements (CAAT-box). The association of allelic changes, **b** 35181691; **c** 35181026; **d** 35179748; **e** 35179634; **f** 35179572; **g** 35177247; with SOC is depicted using box plots

To know the effect of these SNPs on SOC, we performed single SNP association tests for the 55 SNPs among the 290 accessions with different SOCs. The test showed that six SNPs, namely T/G of ChrC07_35,181,691, C/T of ChrC07_35,181,026, C/T of ChrC07_35,179,748, A/T of ChrC07_35,179,634, A/G of ChrC07_35,179,572 and A/G of ChrC07_35,177,247 on the 5′-end regulatory region of *BnaC07g30920D* were significantly (*p* < 0.05) associated with SOC (Fig. 2). Any nucleotide change in the six positions led to a 4.7–6.2% reduction of SOCs (Fig. 2). We also measured the effects of other 4 SNPs in the coding sequence and intron regions. Significant effects were not identified, therefore, we only presented the functional SNPs in Fig. 2. We also showed the effects of these SNPs in Figure S4. In order to identify *cis*-element in the variation site, we BLAST the nucleotide sequence 5 Kb upstream to the start codon in the databases PlantProm (http://linux1.softberry.com/berry.phtml?topic=plantprom&group=data&subgroup=plantprom) and AGRIS (https://agris-knowledgebase.org/), and identified the SNPs such as T/G (ChrC07_35,181,691) and A/T (ChrC07_35,179,634) locating in the CAAT-box, which associated with SOC changes.

**Fig. 3:**
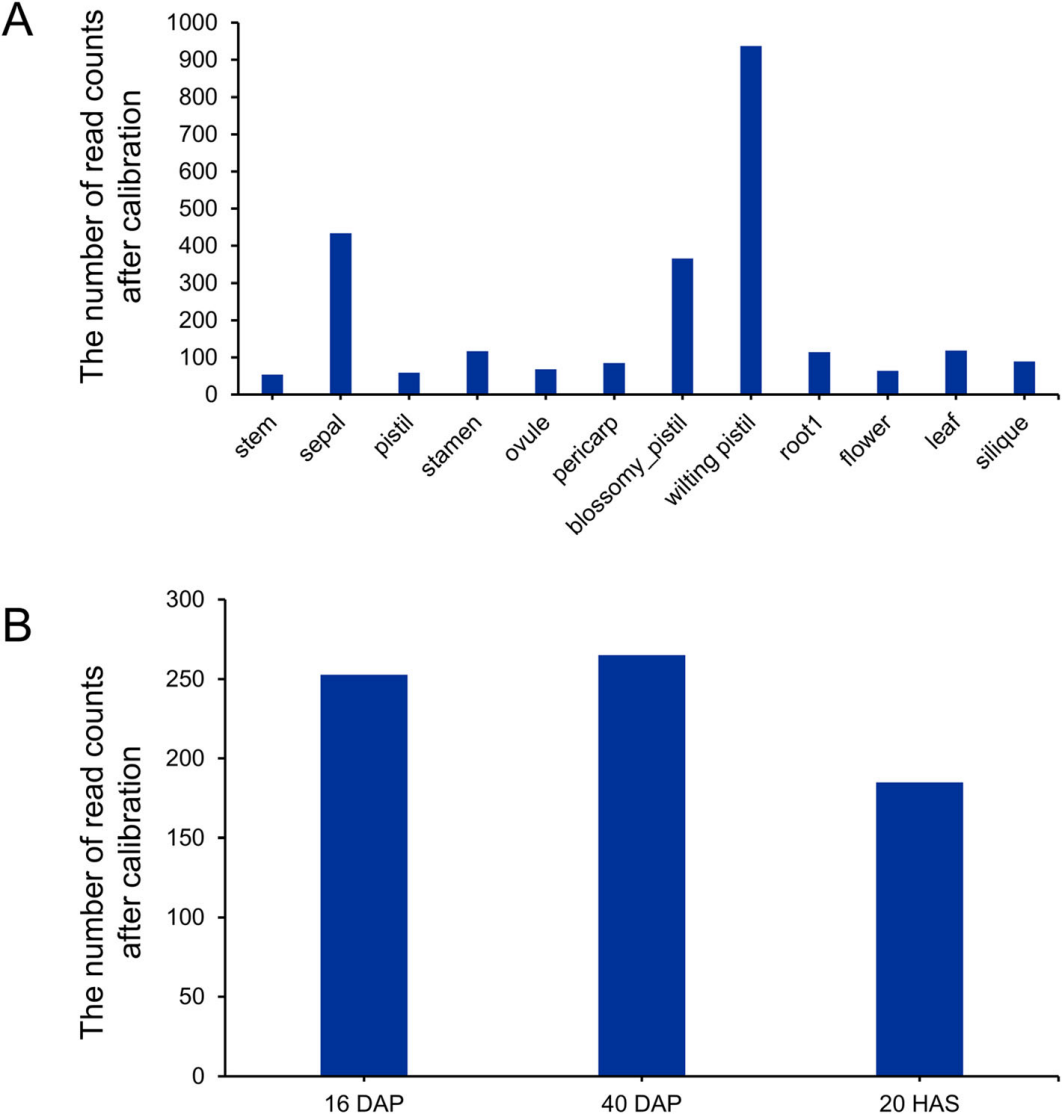
Expression analysis of *BnaC07g30920D*. **a** The expression values of *BnaC07g30920D* in 12 different tissues. **b** The expression values of *BnaC07g30920D* at 16 DAP, 40 DAP of seeds and in germinating seeds 20 HAS

The expression of *BnaC07g30920D* was analyzed based on two transcriptome datasets (Fig. 3). The highest transcriptional levels of *BnaC07g30920D*, which were indicated by the number of read counts after calibration, were observed in pistils and sepals (Fig. 3a). *BnaC07g30920D* was significantly expressed (RPKM> 1) in the seeds at 16 DAP, 40 DAP and in germinating seeds 20 HAS (Table S6). Relative to the 20-HAS germinating seeds, the 16-DAP and 40-DAP seeds had nearly 1.5 folds higher transcriptional level of *BnaC07g30920D* (Fig. 3b, Table S6), indicating high expression of *BnaC07g30920D* during seed maturation.

### In silico analysis of *BnPTLs* in rapeseed genome

**Fig. 4:**
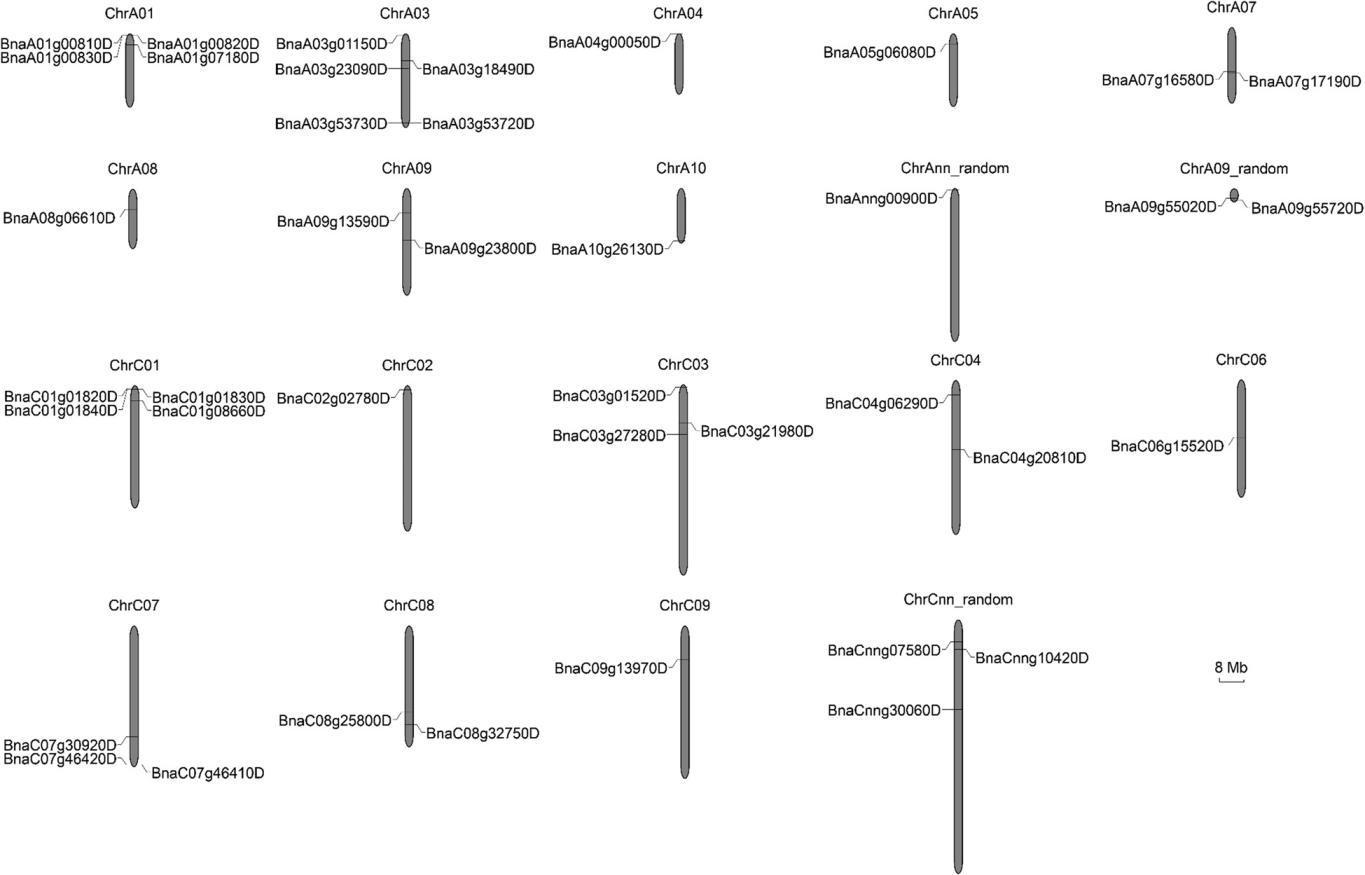
Genomic distribution of *BnPTLs* on *B. napus* chromosomes. Chromosome number is indicated at the top of each chromosome. ‘A09_random’ represents the scaffold which is unmapped on chromosome A09. ‘Ann_random’ and ‘Cnn_random’ represent the unmapped scaffolds in A and C subgenome, respectively. The sequence lengths of every chromosome and the locations of *BnPTLs* are indicated by the scale bar representing 8 megabases (Mb)

Seed oil content is a typical quantitative trait that is controlled by the interaction between numerous major and/or minor genes and environmental factors. In order to excavate potential PTLs that would decompose seed lipid, we performed an in silico analysis to demonstrate the overall distribution of *BnPTLs* across the rapeseed genome (‘Darmor-*bzh*’, V4.1). An InterPro search identified a total of 40 genes containing IPR002641, which were annotated as *PTLs*. Information such as locus ID, genomic sequence length, coding sequence (CDS) and protein size of putative *PTLs* as well as the number of their exons and introns were provided in Table S4. The sizes of genomic sequences, CDSs and proteins of the *BnPTLs* ranged from 1411 to 8211 bp, 873 to 4278 bp, and 290 to 1425 aa, respectively (Table S4). The 40 *BnPTLs* were distributed equally on A subgenome and C subgenome (Fig. 4). Except for four *BnPTL*s (*BnaAnng00900D*, *BnaCnng07580D*, *BnaCnng10420D* and *BnaCnng30060D*) distributed on random chromosomes, the remaining 36 *BnPTLs* were mapped on chromosomes A01-A10 and C01-C09. Notably, there were no *BnPTLs* located on chromosomes A02, A06 and C05 (Fig. 4). There was the largest number of *BnPTLs* (5 out of 40) located on Chromosome A03, whereas, there was merely one *BnPTL* homolog which located on chromosomes A04, A05, A08, A10, C02, C06 and C09 (Fig. 4). Nearly half (19 in 40) of *BnPTLs* located at ends of the chromosomes (Fig. 4).

**Fig. 5:**
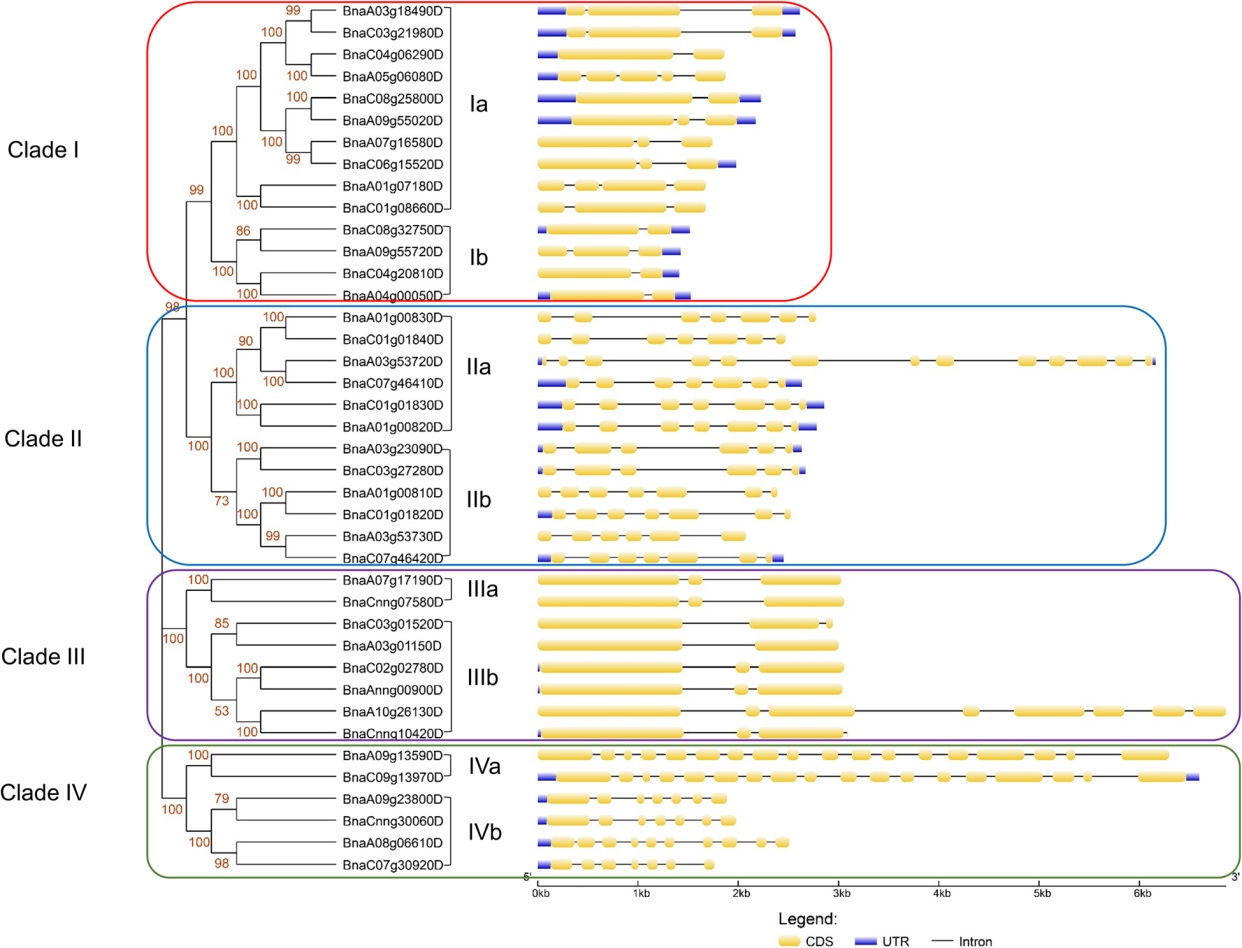
Unrooted phylogenetic tree and gene structure profile of *BnPTLs*. The neighbor-joining (NJ) unrooted tree was constructed based on the multiple alignments of *BnPTL* protein sequences by using ClustalW program with 1000 bootstrap replicates. Clades and subclades are labeled at the left and right of the tree, respectively. The red numbers adjacent to every branch represent the bootstrap values. The gene structure profile located at the right of the tree is depicted with coding sequences (CDS), introns and untranslated regions (UTR)

Gene structure analysis showed that the number of exons of *BnPTLs* ranged from two to eighteen with 5.6 as average. 12 (30%) and 11 (27.5%) *BnPTLs* had three and seven exons interrupted by two and six introns, respectively (Table S4, Fig. 5). The rest of the genes had 2 exon (6 genes), 4 exons (2 genes), 5 exons (1 gene, *BnaA05g06080D*), 6 exons (3 genes), 8 exons (1 gene, *BnaA10g26130D*), 10 exons (1 gene, *BnaA08g06610D*), 13 exons (1 gene, *BnaA03g53720D*) or 18 exons (2 genes), respectively.

To elucidate the evolutionary relationship of *BnPTLs*, we carried out a phylogenetic analysis of *BnPTLs*. In brief, *BnPTLs* could be divided into four clades (I-IV) (Fig. 5). The Clade I, Clade II, Clade III and Clade IV contained 14, 12, 8 and 6 *BnPTL* homologs, accounting for 35, 30, 20 and 15% of the total *BnPTLs*, respectively (Table S4, Fig. 5). There were significantly more *BnPTL* exons in genes of Clade II and Clade IV (8.5 on average) than those in Clade I and Clade III (3.1 on average) (Table S4, Fig. 5).

For any gene family, the conserved sequences and domains are related to their functions. In order to better understand the biological function of *BnPTLs,* we analyzed the conserved domain of *BnPTL*s. The catalytic regions consisting of DGGGXX, elements for phosphate- or anion-binding, and GXSXG which is the esterase box, were built up with 20–30 amino acids and length of the sequences varied among different clades, suggesting various biological functions of the *BnPTL* homologs (Table 2).

**Table 2:** Catalytic centers of patatin-like lipases in *B. napus*

Clade	Sub-clade	Anion binding box	Esterase box
Clade I	Ia	LSIDGGGMRG	VAxGSGxGGxxx
	Ib	LSIDGGGTTxxx	DIVAGTGIGGxL
Clade II		LSxDGGGxRGxx	DVxxGTSTGGLxx
Clade III		LLLSGGASLGAGAFH	RIIAGSSVGSxx
Clade IV	Iva	ILTMDGGGMKGLA	LICGTSTGGMLA
	Ivb	GFSFSAAGLLFPY	DTTPLAGASAGAxVCA

Note: The underlined letters indicate regions of the highest homology

### Expression patterns of *BnPTLs*

**Fig. 6:**
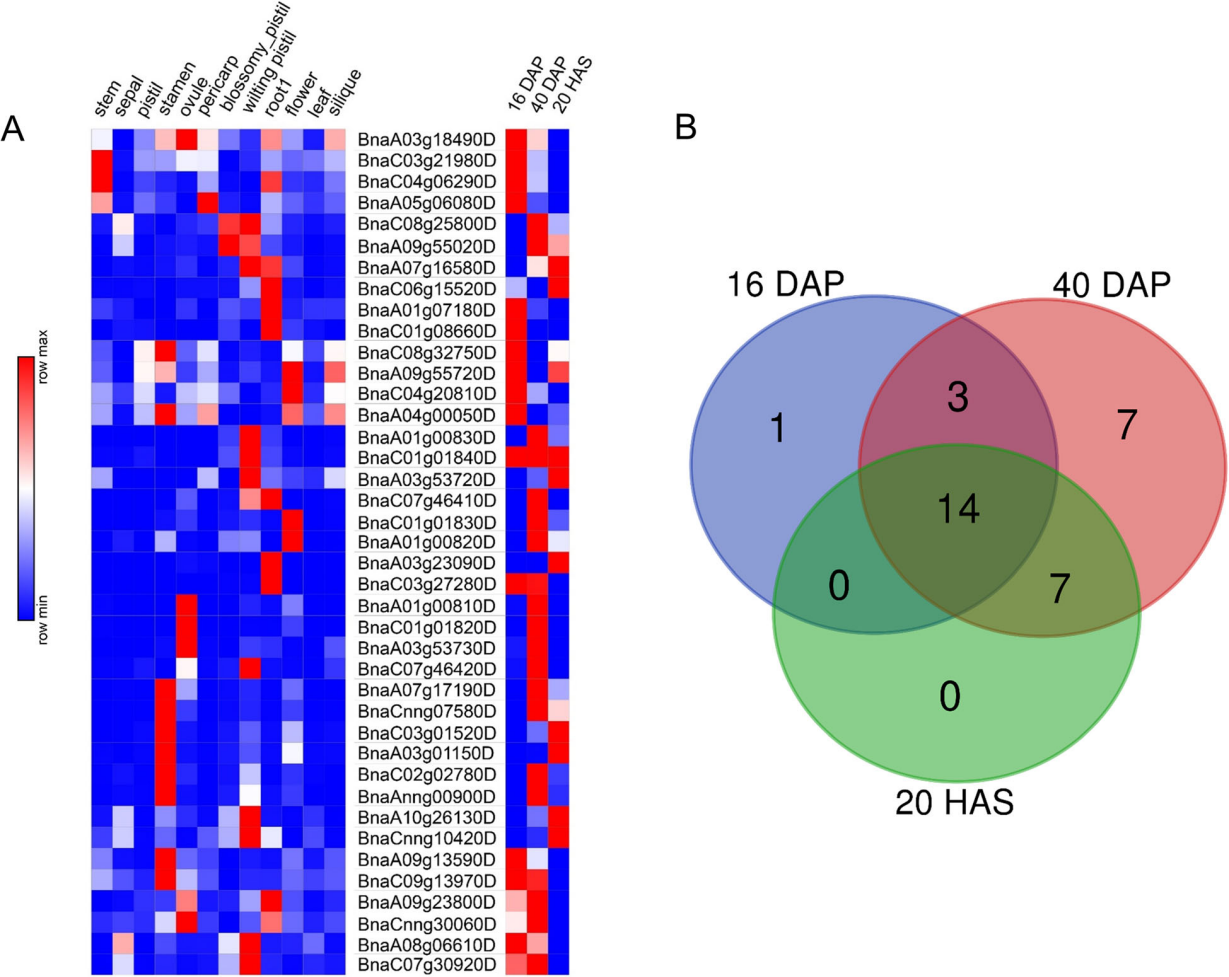
Expression patterns of *BnPTLs* based on 2 transcriptome data sets. **a** The expression heatmap of *BnPTLs*. The expression heatmap of *BnPTLs* in 12 different tissues is at the left of the gene IDs and the heatmap at different seed developmental stages is at the right of the gene IDs. The expression levels are shown by the color. **b** Venn diagram of 40 *BnPTLs* expressed at different seed developmental stages

The expression pattern of *BnPTLs* can give us additional insight into their biological functions. To determine the expression patterns of *BnPTLs* in different tissues, transcriptome data from 12 tissues of the *B. napus* cultivar ‘ZS 11’ were downloaded [36]. Overall, *BnPTLs* were expressed in all 12 tissues, implying their diverse biological functions (Fig. 6a, Table S5). The highest expression of *BnPTLs* was detected in stamens (366 on average) and wilting pistils (362 on average), while the expression of *BnPTLs* in stems (73 on average), roots (112 on average) and leaves (52 on average) were relatively low, indicating that *BnPTLs* were expressed preferentially in reproductive tissues. Among 40 *BnPTLs*, extremely significant expression of some genes such as *BnSDP1* (*BnaCnng10420D*) in wilting pistils (1991) and *BnSDP1-Like* (*BnSDP1L*, *BnaA07g17190D*) in stamens (984), could be observed, suggesting their potential roles in these tissues (Fig. 6a, Table S5).

The *BnPTLs* which expressed in seeds may affect SOC. To determine the expression pattern of *BnPTLs* at different developmental stages of seeds, we analyzed the RNA-Seq transcriptome data from seeds of 16, 40 DAP and 20 HAS. There were a total of 31 *BnPTLs* that were significantly expressed (RPKM> 1) in 40-DAP seeds (Fig. 6, Table S6). In contrast, 18 and 21 *BnPTLs* were expressed in 16-DAP seeds and the 20-HAS germinating seeds, respectively. Notably, 14 *BnPTLs* were significantly expressed in seeds of all three developmental stages (Fig. 6b, Table S6). *BnaA05g06080D* was only expressed in the 16-DAP seeds, whereas, there were seven genes only expressed in 40-DAP seeds. The expression of twelve genes was upregulated in the 40-DAP seeds relative to the 16-DAP seeds (defined by log2 ^(expression folds)^ > 1). On the other hand, the expression of six genes was downregulated in the 40-DAP seeds relative to the 16-DAP seeds (defined by log2 ^(expression folds)^ < 1) (Fig. 6, Table S6). These results suggested that most *BnPTLs* had higher expression at the late stage of seed development.

## Discussion

Crop based oils are valuable agricultural products, and SOC is an important trait of oil crops. Elevating SOC was successfully achieved through traditional breeding and genetic manipulation of genes involving lipid biosynthesis [37–39]. In rapeseed, SOC usually first reaches a peak followed by a decline at the late maturity stage [10]. The SOC reduction at the late maturity stage results from two aspects. First, the expression of genes involved in oil synthesis is significantly decreased. Second, a large number of genes involved in oil degradation are expressed and active at this stage [1]. A recent study reported that SOC of rapeseed is affected by the germination potential of premature developing seeds. The biological significance of the oil content reduction in premature seeds could be the supply of energy and carbon skeleton for the seeds which are going to initiate a next life cycle [12, 40]. Anyway, the decomposition of synthesized lipids during seed maturation eventually led to the reduction of final SOC and downgraded seed quality. Studies showed that *Seed Fatty Acid Reducers* (*SFARs*), namely *SFAR1* to *SFAR5* belonging to the GDSL family were involved in the decomposition of lipids in developing seeds of *Arabidopsis thaliana*, and thus reduced SOC and changed fatty acid composition [41]. More recently, *BnSFAR1* and *BnSFAR4* were successfully silenced by random chemical inducement (Targeting Induced Local Lesions IN Genomes, TILLING), and *BnSFAR4* and *BnSFAR5* were edited by CRISPR-Cas9 mediated technology. Mutant lines with up to 12.1% higher SOC by TILLING and 14.5% higher SOC by CRISPR-Cas9 were created [15]. In this study, we performed GWAS in a genetic population to associated significant SNPs with acting genes underlying a mapping interval which contained 34 genes. We selected the *PTL* (*BnaC07g30920D*) as a candidate gene, which might involve in degrading lipids. We excluded the other 33 genes either from their annotations (Table S2) or by calculating the SNP effects as did with the *BnPTL*. We show that single nucleotide mutations at *BnaC07g30920D*, a *patatin-like lipase* gene, resulted in significant SOC reduction in rapeseed (Fig. 2). Although this *PTL* and *SFARs* might have similar biological function, they were likely to be regulated by different upstream regulators. *SFARs* were regulated by gibberellin signaling [41], whereas *PTL* remained little understood.

For better exploring potential SOC reducers of the PTL family, in silico analysis was performed to unveil the overall distribution of the genes across the polyploid rapeseed genome. Among 40 *BnPTLs*, more than 75% were expressed at 40 DAP (Fig. 6, Table S6), indicating that *BnPTLs* preferentially function at the late stages of seed development. The time-biased expression pattern of *BnPTLs* was in line with the period of SOC decrease. There were seven *BnPTLs* that were exclusively expressed in 40-DAP seeds and 20-HAS germinating seeds (Fig. 6b, Table S6), implying their potential role in oil degradation at these two stages.

As a member of PTL family, SUGAR-DEPENDENT1 (SDP1) is a TAG lipase which is present in many plants such as *Arabidopsis thaliana*, *Brassica napus*, *Jatropha curcas* and *Glycine max*. Many reports have shown that SDP1 was involved in TAG degradation and the silence of *SDP1* could enhance oil content in seeds and other tissues [22, 23, 42–44]. However, the association between the allelic variation of *BnSDP1* and SOC was not identified in our GWAS. SOC is a typical quantitative trait that is controlled by the interaction between numerous genes and environmental factors. Our field experiments were carried through in two locations with 400 KM in distance. It could not be excluded that the effect of *BnSDP1* could be detected given an environment that could be more different.

In general, lipases are acyl-hydrolases hydrolyzing the ester bond of mono-, di- and triglycerides (TAGs) into fatty acids and glycerol [45]. Lipases can be divided into three subfamilies according to the structural features of their conserved domains. The largest lipase subfamily contains a conserved signature motif (GXSXG) and their catalytic triad consists of the catalytic serine (S) in this conserved motif, a histidine (H) and either an aspartate (D) or a glutamate (E) located downstream in the sequence [45, 46]. GDSL-type lipases, which belong to the SGNH hydrolase superfamily, harbor a GDSL sequence motif in sequence block 1 adjacent to the N-terminus instead of the GXSXG motif. The catalytic triad comprises the serine (S) in GDSL motif, the histidine (H) within sequence block 5 and the aspartate (D) 3 amino acids ahead of the H (DxxH) [45, 47, 48]. Patatin is a lipolytic acyl-hydrolase that is abundant in potato tubers [16]. The biggest structural difference between patatin and other lipases is that its catalytic domain consists of a Ser-Asp dyad. There are 222 GDLS family genes but only 40 PTL family genes in the rapeseed genome (Fig. 4). Enzymes with the patatin catalytic domain, namely PTLs, are widely present in yeast, bacterial, animal and plant. Most of TAG lipases belong to the PTL family, such as TGL3 (yeast), ATGL (mammal) and SDP1 (plant) [18, 45, 49]. The broad substrate specificity of PTLs suggests their multiple biological functions. For example, *DENSE AND ERECT PANICLE3* (*DEP3*), a *PTL* in rice, was found to play a significant role in the formation of vascular bundles in rice [50]; *pPLAIIIδ* had a marked impact on auxin-responsive cell morphology and organ size in *Arabidopsis* and *B. napus* [51]; *VdPLP*, a *PTL* in *Verticillium dahliae*, was involved in cell wall integrity and required for pathogenicity [52]. We classified the 40 *BnPTLs* in rapeseed into four clades in light of their gene structure and nucleotide sequence similarity. However, the detailed functions of most members of the *BnPTL* family remain to be explored.

In a recent study, we reported the resequencing of 991 accessions of a world-wide rapeseed (*Brassica napus*) germplasm collection. A total of 5.56 million SNPs and 1.86 million indels were identified by mapping the reads to a reference genome (‘Darmor-*bzh*’) [29]. In this study, we established a gene bank including only 290 accessions based on genetic diversity analysis of the 991 accessions. These 290 accessions stand for more than 97% genetic polymorphism of the 991-accessions population in terms of SNPs and InDels, the population was small though. Relative to previous GWASs in rapeseed [29, 30, 53–55], the present study applied a much smaller number of genetic accessions which was easily treated for field replicates. However, the 2,705,480 SNPs, which were very high in number, allow a very powerful identification of tightly associated genes. The results enrich our knowledge about the SOC formation in rapeseed and facilitate the future study in functional characterization of *BnPTL* genes.

## Conclusions

Patatin-like lipases (PTLs) are widely present in plants and the functions of most PTLs are little understood. In the present study, a *BnPTL* gene was identified to be associated with SOC using a GWAS on SOC in a collection of 290 core germplasm accessions. A total of 40 *BnPTL* genes were characterized and divided into four clades, evenly distributed on the A and C subgenomes of *B. napus*. Moreover, RNA-seq analysis unveiled that *BnPTLs* were preferentially expressed in reproductive tissues especially maturing seeds. These results enrich our knowledge about the SOC formation in rapeseed and provide more information for future study in functional characterization of *BnPTL* genes in *B. napus*.

## Methods

### Plant materials and growth conditions

The 290 core accessions of rapeseed germplasm for GWAS were selected from a worldwide germplasm collection of 991 accessions [29], which were also used to unveil new genes involved in leaf trichome formation in rapeseed in our previous studies [28]. A part of the accessions were acquired from the Leibniz Institute of Plant Genetics and Crop Plant Research (https://gbis.ipk-gatersleben.de/gbis2i/faces/index.jsf) in Gatersleben, Germany, and the rest were from the Provincial Key Laboratory of Crop Gene Resources of Zhejiang University in China. The information about their ID, country origin and SOC of each accession was listed in Table S1. They were grown in the experimental field of Changxing Agricultural Experiment Station of Zhejiang University (30°02′N and 119°93′E) in 2017 and the Experimental Farm of Jinhua Academy of Agricultural Sciences (29°05′N and 119°38′E) in 2018, respectively.

### Seed oil content measurement and phenotypic analysis

Mature seeds of the 290 core accessions were harvested for SOC measurement. SOCs were determined using near-infrared spectroscopy (ANTARIS II, Thermo Scientific™, America). Three biological replicates of each accession were measured. Phenotypic analysis including the mean, standard deviation, correlation coefficient, and minimum and maximum values of SOC from 290 accessions were calculated and analyzed using SPSS (http://www.ibm.com/cn-zh/analytics/spss-statistics-software; V21.0.0.0). Variations in SOC were analyzed by analysis of variance (ANOVA).

### Genome-wide association study

A total of 2,705,480 high-quality SNPs (MAF > 0.05, missing rates< 0.5) among the 290 core accessions were extracted and used for GWAS. The K-value, which represents the genetic relations between samples, was calculated by Plink software (http://www.cog-genomics.org/plink2; V1.9) [56]. TASSEL software (http://www.maizegenetics.net/tassel) [57] with an Efficient Mixed-Model Association eXpedited (EMMAX) was used to detect the associations. The *p*-value of each SNP was calculated with -log_10_^*p*^ > 5 as the suggestive threshold. Seventy-five kilobase sequence regions adjacent to significantly associated SNPs were searched for associated genes. The R package “LDheatmap” (http://cran.r-project.org/web/packages/LDheatmap/index.html; V0.99) was used to describe the degree of correlation between significantly associated SNP and candidate genes.

### Identification of *PTLs* in *B. napus*

The full genome sequence, protein sequences and their locations of *B. napus* (‘Darmor-*bzh*’, V4.1) were retrieved from *Brassica napus* Genome Browser in GENOSCOPE (http://www.genoscope.cns.fr/brassicanapus/) [58]. Protein sequences were used as queries to search against InterPro database (http://www.ebi.ac.uk/interpro/search/sequence/; V79.0) [59] with the default parameters. In total, 40 protein sequences with IPR002641 (Patatin-like phospholipase domain) were identified. The distribution of *BnPTLs* across the rapeseed genome was visualized using MapGene2Chromosome V2.0 (http://mg2c.iask.in/mg2c_v2.0/).

### Search for *cis*-elements

We BLAST the nucleotide sequence 5 Kb upstream to the start codon in the databases PlantProm (http://linux1.softberry.com/berry.phtml?topic=plantprom&group=data&subgroup=plantprom) [60] and AGRIS (https://agris-knowledgebase.org/) [61].

### Phylogenetic analysis of BnPTLs and conserved domain search

Multiple alignments were performed for *BnPTL* protein sequences using ClustalW of the Molecular Evolutionary Genetics Analysis (MEGAX) program [62]. The unrooted phylogenetic tree was constructed using the neighbor-joining (NJ) method (the Jones–Taylor–Thornton model) with 1000 bootstrap replicates based on the alignment of *BnPTL* protein sequences in MEGAX. The gene structure and phylogenetic tree were combined using Gene Structure Display Server V2.0 (GSDS 2.0; http://gsds.cbi.pku.edu.cn/) [63]. The conserved domains of *BnPTLs* were searched based on the previous study [18].

### RNA-seq analysis

Transcriptome data from 12 tissues of the *B. napus* cultivar ‘ZS 11’ which was released in the previous study [36] were retrieved from National Center for Biotechnology Information (NCBI; http://www.ncbi.nlm.nih.gov/) (ID: PRJNA394926). DESeq2 R package (http://bioconductor.org/; V3.11) [64] was applied for expression analysis. Transcriptome data of seeds at 16 days after pollination (DAP), 40 DAP and germinating seeds 20 h after sowing (HAS) were obtained from the previous study [1]. Expression values of these two transcriptome datasets were represented by the number of read counts after calibration. In order to better compare the expression levels in different periods, we also calculated the reads per kilobase per million mapped reads (RPKM) of the latter dataset. The heatmap of *BnPTLs* was constructed with Morpheus (http://software.broadinstitute.org/morpheus).

## Supplementary Information

**Additional file 1: Figure S1.** The frequency distribution for SOC of 290 rapeseed accessions in CX (A) and JH (B).

**Additional file 2: Figure S2.** Manhattan plots of GWAS for CX (A) and Jinhua (B). The blue line represents a significant threshold (−log_10_*p* = 5). The black triangle indicates the SNP, ChrC07_35249208, associated with *BnaC07g30920D*.

**Additional file 3: Figure S3.** Quantile-quantile (Q-Q) plots of GWAS for CX (A) and JH (B). The Y-axis is the observed negative base 10 logarithms of the *P*-values and the X-axis is the expected observed negative base 10 logarithms of the *P*-values under the assumption that the *P*-values follow a uniform (0,1) distribution.

**Additional file 4: Figure S4.** The association of allelic changes in the CDS region and intron of *BnaC07g30920D* with SOC. ChrC07_35175173 (A) and ChrC07_35175214 (B) were located in introns, while ChrC07_35175691 (C) and ChrC07_35176144 (D) were located in the CDS region.

**Additional file 5: Table S1.** The accession codes, names, origins of the 290 genetic materials and their SOC at two experimental sites.

**Additional file 6: Table S2.** The SNPs associated with SOC on Chr.C07 and associated gene IDs on Chr.C07.

**Additional file 7: Table S3.** The SNPs across *BnaC07g30920D*.

**Additional file 8: Table S4.** The genome-wide analysis of 40 *BnPTLs* in rapeseed.

**Additional file 9: Table S5.** The expression values (indicated by the number of read counts after calibration) of *BnPTLs* in 12 different tissues of rapeseed.

**Additional file 10: Table S6.** The expression values (indicated by RPKM and the number of read counts after calibration) of *BnPTLs* at different seed developmental stages of rapeseed.

## Data Availability

The raw reads of the rapeseed accessions used in this study have been deposited in the public database of National Center of Biotechnology Information under SRP155312 (https://www.ncbi.nlm.nih.gov/sra/SRP155312). SNPs among the genetic accessions can be conveniently retrieved at BnaSNPDB (https://bnapus-zju.com/bnasnpdb/). The supporting transcriptome datasets are available at NCBI Bioproject repository, accession number: PRJNA394926.

## Abbreviations

ANOVA: Analysis of variance
ATGL: Adiponutrin
*BnPTL*: *Brassica napus patatin-like lipase*
CDS: Coding sequence
CV: Coefficient of variation
CX: Changxing
DAP: Days after pollination
DEP3: DENSE AND ERECT PANICLE3
EMMAX: Efficient Mixed-Model Association eXpedited
FA: Fatty acid
GWAS: Genome-wide association study
HAS: Hours after sowing
JH: Jinhua
LD: Linkage disequilibrium
LEC1: LEAFY COTYLEDON1
MEGAX: Molecular Evolutionary Genetics Analysis
NJ: Neighbor-joining
PTL: Patatin-like lipase
Q-Q: Quantile-quantile
QTL: Quantitative trait locus
RPKM: Reads per kilobase per million mapped reads
SDP1: SUGAR-DEPENDENT1
SFAR: Seed Fatty Acid Reducer
SNP: Single nucleotide polymorphism
SOC: Seed oil content
TAG: Triacylglycerol
TF: Transcription factor
TILLING: Targeting Induced Local Lesions IN Genomes
