# Supplementary figures and images for: Genome-wide association study reveals a patatin-like lipase relating to the reduction of seed oil content in Brassica napus
Source: BMC Plant Biol. 2021 Jan 6;21:6. doi: 10.1186/s12870-020-02774-w (PMC7788869; doi:10.1186/s12870-020-02774-w)

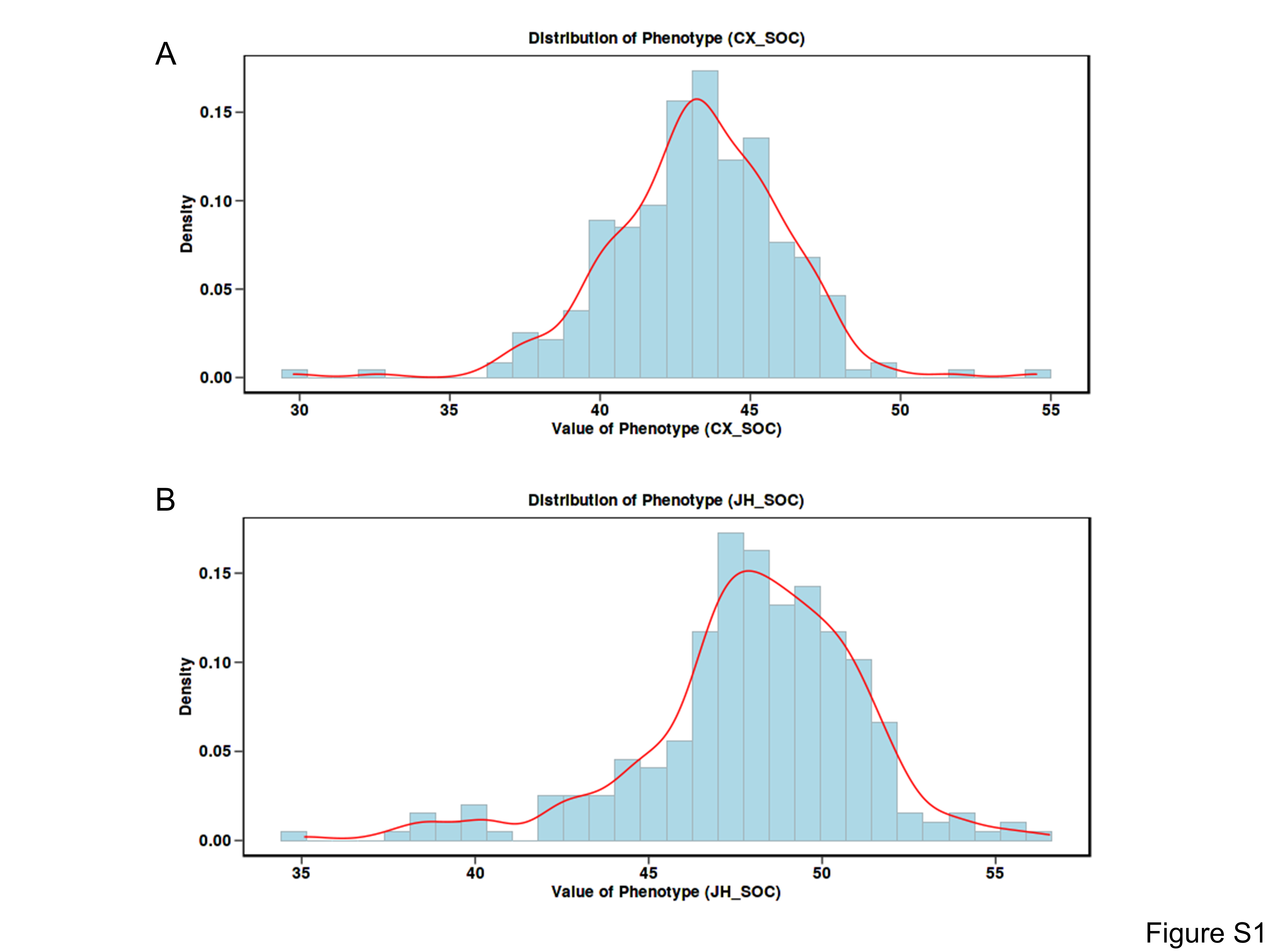

Supplement: Supplementary file 1 — Additional file 1: Figure S1. The frequency distribution for SOC of 290 rapeseed accessions in CX (A) and JH (B). [file 12870_2020_2774_MOESM1_ESM.tif]

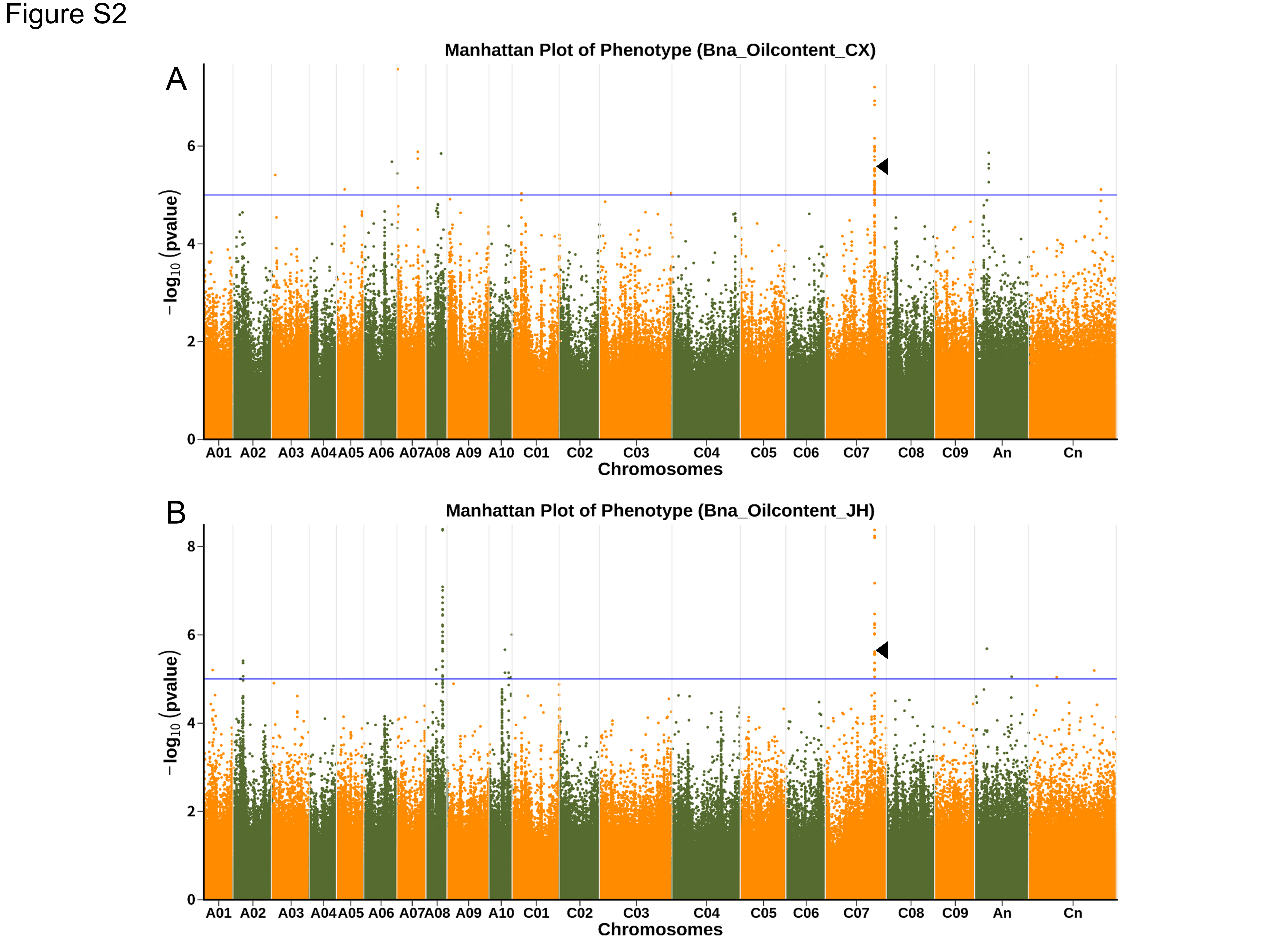

Supplement: Supplementary file 2 — Additional file 2: Figure S2. Manhattan plots of GWAS for CX (A) and Jinhua (B). The blue line represents a significant threshold (−log10p = 5). The black triangle indicates the SNP, ChrC07_35249208, associated with BnaC07g30920D. [file 12870_2020_2774_MOESM2_ESM.tif]

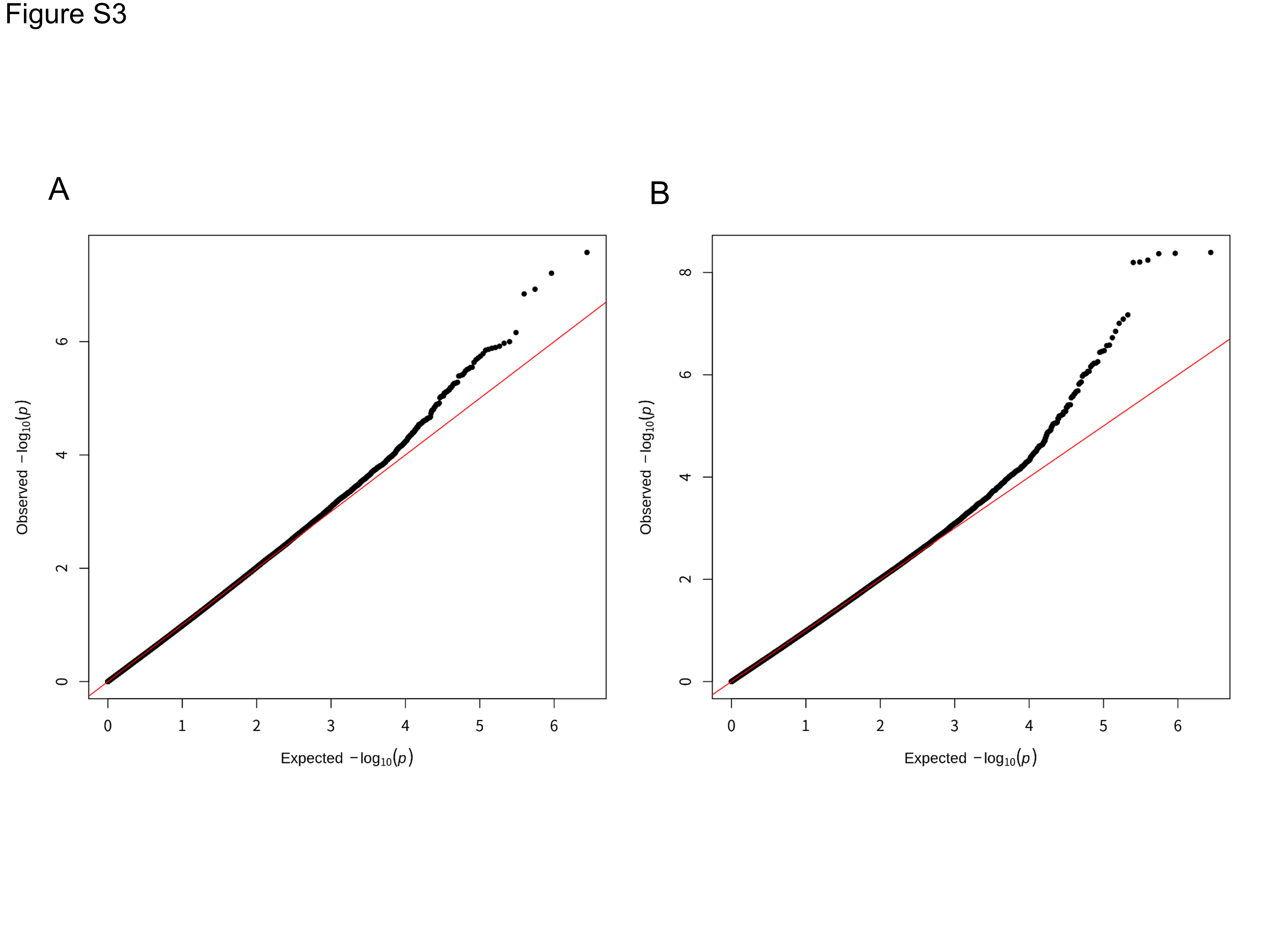

Supplement: Supplementary file 3 — Additional file 3: Figure S3. Quantile-quantile (Q-Q) plots of GWAS for CX (A) and JH (B). The Y-axis is the observed negative base 10 logarithms of the P-values and the X-axis is the expected observed negative base 10 logarithms of the P-values under the assumption that the P-values follow a uniform (0,1) distribution. [file 12870_2020_2774_MOESM3_ESM.tif]

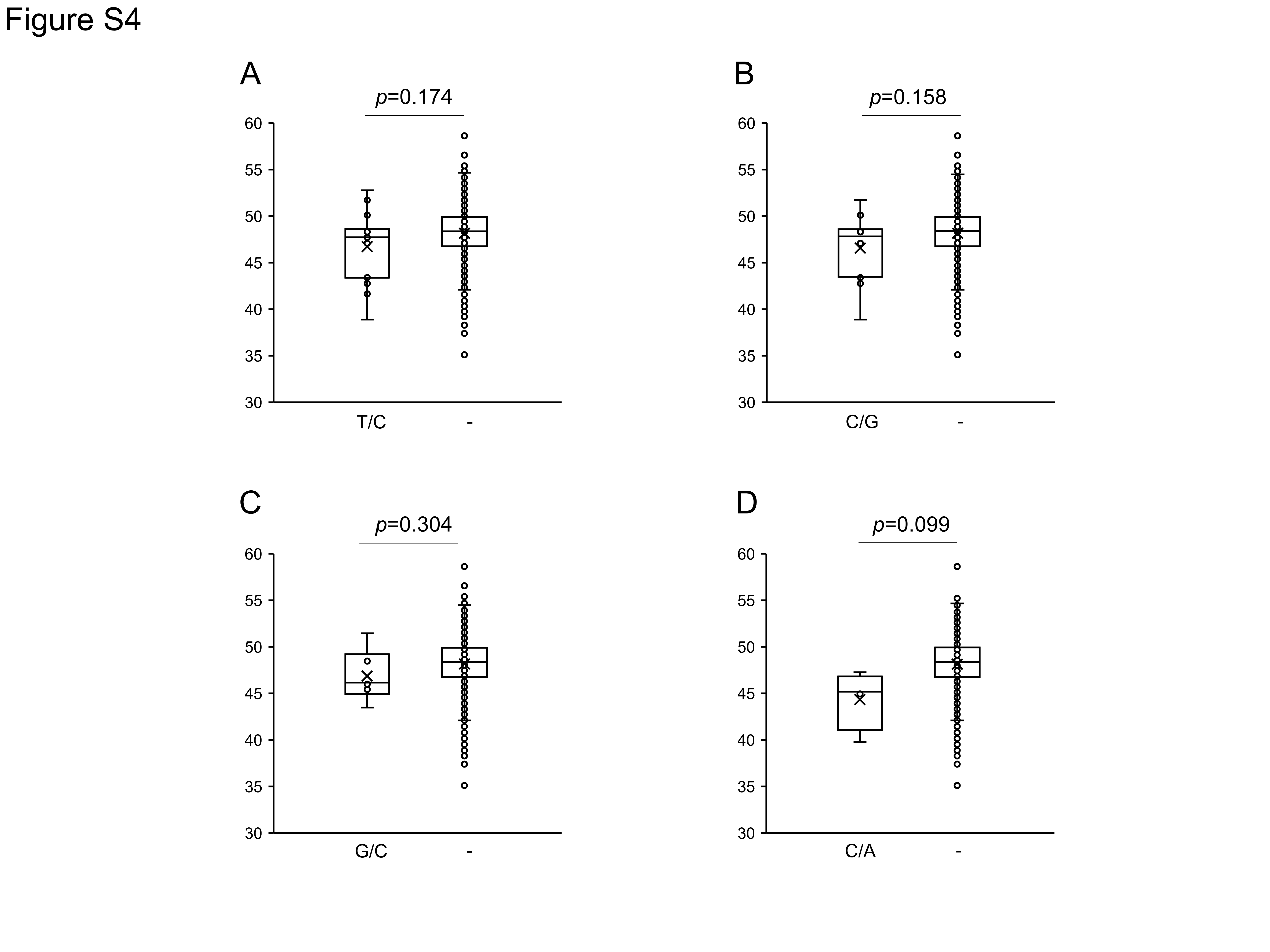

Supplement: Supplementary file 4 — Additional file 4: Figure S4. The association of allelic changes in the CDS region and intron of BnaC07g30920D with SOC. ChrC07_35175173 (A) and ChrC07_35175214 (B) were located in introns, while ChrC07_35175691 (C) and ChrC07_35176144 (D) were located in the CDS region. [file 12870_2020_2774_MOESM4_ESM.tif]
